# SGLT2 Inhibitors as a Novel Therapeutic Strategy in SIADH-Induced Hyponatraemia: Emerging Evidence and Clinical Implications

**DOI:** 10.3390/jcm15062119

**Published:** 2026-03-10

**Authors:** Neena Kamran, Misbah Mohammad

**Affiliations:** Department of Diabetes and Endocrinology, Mid Yorkshire Teaching NHS Trust, Wakefield WF1 4DG, UK; misbah.mohammad@nhs.net

**Keywords:** SIADH, hyponatraemia, SGLT2 inhibitors, vasopressin, empagliflozin, dapagliflozin, electrolyte disorders

## Abstract

Syndrome of inappropriate antidiuretic hormone secretion (SIADH) is a leading cause of euvolaemic hyponatraemia and remains challenging to manage due to limitations of existing therapies, including poor adherence to fluid restriction and safety concerns associated with vasopressin receptor antagonists and demeclocycline. Recent mechanistic and clinical evidence suggests that sodium–glucose cotransporter-2 (SGLT2) inhibitors may offer a novel therapeutic approach by promoting osmotic diuresis and increasing electrolyte-free water clearance. This narrative review synthesises current pathophysiological understanding and emerging clinical evidence regarding the role of SGLT2 inhibitors in SIADH-related hyponatraemia. We examine how their proximal tubular mechanism differs from conventional therapies such as loop diuretics, vaptans, and salt supplementation, and evaluate evidence from recent randomised and crossover trials demonstrating improved serum sodium and enhanced water excretion with empagliflozin and dapagliflozin. SGLT2 inhibitors represent a physiologically rational and potentially safer alternative in selected patients with SIADH, particularly where fluid restriction is poorly tolerated or ineffective. Although further large-scale studies are required to define their optimal positioning within treatment algorithms, current evidence supports their potential role as an emerging therapeutic option in SIADH management.

## 1. Introduction

The syndrome of inappropriate antidiuretic hormone secretion (SIADH) represents the most common cause of euvolaemic hyponatraemia and is frequently encountered in both inpatient and outpatient clinical practice. It is characterised by persistent secretion or action of arginine vasopressin (AVP), also known as antidiuretic hormone (ADH), despite normal or low plasma osmolality, resulting in impaired renal free water excretion, dilutional hyponatraemia, and inappropriately concentrated urine. Hyponatraemia associated with SIADH is not merely a biochemical abnormality but is linked to significant clinical consequences, including gait disturbance, falls, cognitive impairment, osteoporosis, and increased mortality, particularly in elderly and hospitalised populations [1,2,3,4,5,6].

The aetiology of SIADH is heterogeneous and includes malignancies—most notably small-cell lung carcinoma—pulmonary disorders such as pneumonia, central nervous system pathologies including stroke and meningitis, and a wide range of medications, including selective serotonin reuptake inhibitors, antipsychotics, and anticonvulsants. In many cases, SIADH persists chronically, requiring long-term management strategies. Despite its prevalence and clinical significance, optimal treatment remains challenging due to limitations associated with current therapeutic approaches.

Fluid restriction remains the cornerstone of management; however, its effectiveness is often limited by poor patient adherence and unpredictable efficacy, particularly in individuals with high urine osmolality. Pharmacological therapies, including vasopressin receptor antagonists such as tolvaptan and the tetracycline derivative demeclocycline, are effective in selected cases but are constrained by safety concerns, cost, monitoring requirements, and potential toxicity, including hepatotoxicity and nephrotoxicity. Loop diuretics combined with sodium supplementation are also used but are associated with variable outcomes and limited high-quality evidence supporting their widespread use [1,2,3,4,5,6].

Given these limitations, there is increasing interest in therapeutic strategies that directly enhance renal free water clearance through physiologically targeted mechanisms. Sodium–glucose cotransporter-2 (SGLT2) inhibitors, initially developed for the treatment of type 2 diabetes mellitus, have emerged as potential candidates. These agents inhibit glucose and sodium reabsorption in the proximal convoluted tubule, resulting in glucosuria-induced osmotic diuresis, increased urinary volume, and reduction in total body water. Importantly, this mechanism operates independently of vasopressin receptor antagonism and without disruption of the renal medullary concentration gradient, distinguishing SGLT2 inhibitors from conventional therapies.

Beyond their established cardiovascular and renal benefits, emerging mechanistic and clinical evidence suggests that SGLT2 inhibitors may increase electrolyte-free water clearance and improve serum sodium concentrations in patients with SIADH. Randomised controlled trials and mechanistic studies have demonstrated modest but clinically meaningful improvements in serum sodium levels, alongside favourable tolerability profiles. These findings raise the possibility that SGLT2 inhibitors may represent a novel and physiologically rational therapeutic option in the management of SIADH-associated hyponatraemia.

This narrative review aims to provide a comprehensive overview of the pathophysiology of SIADH and the mechanistic basis for the use of SGLT2 inhibitors in this condition. We critically evaluate emerging clinical evidence, compare SGLT2 inhibitors with existing treatment modalities, and discuss their potential role within current treatment algorithms. In addition, we explore safety considerations and highlight key areas for future research to better define the therapeutic positioning of this novel approach [1,2,3,4,5,6].

**Literature Search Strategy:** This narrative review was conducted using a structured literature search of PubMed, MEDLINE, and EMBASE databases up to January 2026. Search terms included combinations of “SIADH”, “hyponatraemia”, “SGLT2 inhibitors”, “empagliflozin”, “dapagliflozin”, and “electrolyte-free water clearance”. Clinical trials, mechanistic studies, and relevant review articles were screened for inclusion based on relevance to SIADH pathophysiology and therapeutic implications. Given the emerging nature of this field, emphasis was placed on randomised controlled trials and mechanistic investigations evaluating serum sodium response and free water clearance.

## 2. Epidemiology and Clinical Impact of SIADH

SIADH represents the most common cause of euvolaemic hyponatraemia and is responsible for a substantial proportion of hyponatraemia cases encountered in clinical practice. Hyponatraemia itself is the most frequent electrolyte abnormality observed in hospitalised patients, affecting approximately 15–30% of inpatients, with SIADH accounting for a significant proportion of these cases. The prevalence is particularly high in elderly populations, reflecting increased exposure to precipitating factors such as malignancy, polypharmacy, and chronic neurological and pulmonary disease [7,8,9,10,11].

Malignancy is one of the most well-recognised causes of SIADH, with small-cell lung carcinoma being the classical example due to ectopic vasopressin production. However, SIADH is also observed in association with other malignancies, including head and neck cancers, gastrointestinal tumours, and lymphomas. Pulmonary conditions such as pneumonia, tuberculosis, and acute respiratory failure can also stimulate vasopressin release. Central nervous system disorders—including stroke, subarachnoid haemorrhage, traumatic brain injury, infections, and neurodegenerative diseases—are another major category of underlying causes.

Medications represent an increasingly important contributor to SIADH, particularly in older adults. Commonly implicated drugs include selective serotonin reuptake inhibitors, serotonin–noradrenaline reuptake inhibitors, antipsychotics, carbamazepine, cyclophosphamide, and certain chemotherapeutic agents. Drug-induced SIADH may develop shortly after initiation or during chronic therapy and is an important reversible cause [7,8,9,10,11].

The clinical consequences of SIADH-related hyponatraemia extend beyond biochemical abnormalities. Even mild chronic hyponatraemia has been associated with impaired cognition, gait instability, increased risk of falls, fractures, and reduced quality of life. In hospitalised patients, hyponatraemia is associated with increased length of stay, higher healthcare costs, and increased mortality.

The burden of chronic SIADH is particularly significant in outpatient settings, where persistent hyponatraemia may require long-term management. However, current treatment options are limited, and many patients remain chronically hyponatraemic despite therapy. This highlights the importance of developing effective and well-tolerated treatment strategies [7,8,9,10,11].

The emergence of SGLT2 inhibitors as potential therapeutic agents is particularly relevant in this context, given their favourable safety profile, oral administration, and physiological mechanism of action. Their potential role in addressing the unmet therapeutic needs of patients with chronic SIADH represents an important area of clinical interest.

## 3. Pathophysiology of SIADH

The syndrome of inappropriate antidiuretic hormone secretion (SIADH) is characterised by impaired renal free water excretion due to persistent secretion or action of arginine vasopressin (AVP), resulting in dilutional hyponatraemia despite normal or increased total body water and clinically euvolaemic status. Under physiological conditions, AVP release from the posterior pituitary is tightly regulated by plasma osmolality and intravascular volume through hypothalamic osmoreceptors and baroreceptors. Even small increases in plasma osmolality stimulate AVP secretion, promoting water reabsorption in the renal collecting duct, whereas suppression of AVP allows excretion of dilute urine and maintenance of osmotic homeostasis [12,13,14,15,16].

In SIADH, this regulatory mechanism becomes uncoupled from osmotic and haemodynamic control. AVP secretion persists inappropriately despite hypotonic plasma, leading to continued water reabsorption and progressive dilution of serum sodium concentration. The resulting hyponatraemia reflects an excess of total body water relative to total body sodium rather than an absolute deficit of sodium.

At the renal level, AVP exerts its effects primarily through binding to vasopressin V2 receptors located on the basolateral membrane of principal cells in the collecting duct. Activation of these receptors stimulates cyclic adenosine monophosphate (cAMP) signalling pathways, resulting in phosphorylation and insertion of aquaporin-2 water channels into the apical membrane. These channels facilitate passive movement of water down the osmotic gradient from the tubular lumen into the hypertonic renal medullary interstitium. Water is subsequently transported into the systemic circulation via aquaporin-3 and aquaporin-4 channels on the basolateral membrane. This process significantly increases water reabsorption independent of solute, thereby reducing electrolyte-free water excretion.

The net effect is an expansion of total body water, which initially leads to mild extracellular fluid volume expansion. However, compensatory physiological mechanisms rapidly restore apparent euvolaemia. These include suppression of the renin–angiotensin–aldosterone system, increased secretion of natriuretic peptides, and enhanced renal sodium excretion. This adaptive natriuresis prevents overt fluid overload but exacerbates dilutional hyponatraemia by further reducing total body sodium relative to water.

The kidney’s ability to excrete free water depends critically on both suppression of vasopressin and adequate solute delivery to the distal nephron. In normal physiology, the generation of dilute urine requires reabsorption of sodium and chloride in the thick ascending limb of the loop of Henle, which creates the corticomedullary osmotic gradient essential for urinary concentration and dilution. In SIADH, persistent vasopressin activity overrides this mechanism by increasing water permeability in the collecting duct, preventing the excretion of dilute urine even when solute transport mechanisms remain intact [12,13,14,15,16,17,18,19,20,21].

Free water clearance is a key physiological parameter in understanding SIADH. It reflects the kidney’s ability to excrete water in excess of solute and is calculated based on urine osmolality relative to plasma osmolality. In SIADH, urine osmolality remains inappropriately elevated, typically exceeding 100 mOsm/kg, indicating impaired free water clearance. This distinguishes SIADH from conditions such as primary polydipsia, where vasopressin suppression allows maximal dilution of urine.

Chronic exposure to elevated vasopressin also induces adaptive changes at the cellular level, including sustained upregulation of aquaporin-2 expression and enhanced water permeability in the collecting duct. These adaptations further reinforce the kidney’s inability to excrete free water and contribute to the persistence of hyponatraemia [12,13,14,15,16].

The pathophysiological hallmark of SIADH is therefore an imbalance between water intake and renal water excretion, driven primarily by inappropriate vasopressin activity. Importantly, the fundamental defect lies not in sodium loss but in impaired excretion of electrolyte-free water [12,13,14,15,16].

This mechanistic understanding provides the physiological rationale for therapeutic strategies aimed at increasing free water clearance. Traditional therapies such as vasopressin receptor antagonists directly block AVP action at the collecting duct, whereas loop diuretics disrupt the renal medullary concentration gradient. In contrast, sodium–glucose cotransporter-2 (SGLT2) inhibitors act upstream in the proximal convoluted tubule by inhibiting sodium and glucose reabsorption. This results in glucosuria-induced osmotic diuresis, increasing tubular fluid volume and enhancing solute delivery to the distal nephron. The increased osmotic load limits passive water reabsorption in the collecting duct, effectively increasing electrolyte-free water excretion despite ongoing vasopressin activity.

This unique mechanism allows SGLT2 inhibitors to reduce total body water through a physiologically targeted process without directly interfering with vasopressin receptors or disrupting the medullary concentration gradient. As a result, they represent a mechanistically distinct approach to correcting dilutional hyponatraemia in SIADH [12,13,14,15,16].

## 4. Limitations of Current Therapies

The management of SIADH remains challenging due to significant limitations associated with existing therapeutic options. Although several treatments are available, each is associated with important drawbacks that limit their effectiveness, tolerability, or safety.

Fluid restriction remains the cornerstone of first-line management. However, its effectiveness is often limited, particularly in patients with high urine osmolality, where the kidney’s ability to excrete free water is severely impaired. In such cases, even strict fluid restriction may fail to achieve meaningful correction of serum sodium. Furthermore, fluid restriction is frequently poorly tolerated by patients, particularly in chronic SIADH, due to persistent thirst driven by underlying pathophysiological mechanisms. Poor adherence is common, reducing its effectiveness in real-world clinical practice.

Vasopressin receptor antagonists, such as tolvaptan, directly target the underlying pathophysiology by blocking vasopressin V2 receptors in the collecting duct, thereby promoting free water excretion. While effective, their use is limited by several important concerns. These include the risk of overly rapid correction of hyponatraemia, which can lead to osmotic demyelination syndrome, a potentially devastating neurological complication. In addition, hepatotoxicity has been reported with prolonged use, requiring careful monitoring of liver function. The high cost of these agents further restricts their widespread use, particularly in outpatient settings.

Demeclocycline represents another treatment option and acts by inducing nephrogenic diabetes insipidus, thereby reducing renal responsiveness to vasopressin. However, its use is limited by delayed onset of action, unpredictable effectiveness, and risk of nephrotoxicity. This is particularly concerning in elderly patients and those with pre-existing renal impairment.

Loop diuretics, often used in combination with oral sodium supplementation, aim to reduce renal concentrating ability and increase free water excretion. However, this approach can be associated with excessive sodium loss, electrolyte disturbances, and volume depletion. The supporting evidence for this strategy is also limited, and its effectiveness is variable.

Urea represents an effective osmotic therapy but is often poorly tolerated due to its unpleasant taste, which can significantly limit patient adherence.

Taken together, these limitations highlight the need for alternative therapeutic strategies that are effective, safe, well-tolerated, and suitable for long-term use. The emergence of SGLT2 inhibitors represents a promising potential solution to address these unmet clinical needs.

## 5. Mechanism of Action of SGLT2 Inhibitors in SIADH

SGLT2 inhibitors act in the proximal convoluted tubule by blocking sodium–glucose cotransport, resulting in glucosuria and osmotic diuresis. The presence of unreabsorbed glucose within the tubular lumen retains water in the nephron and increases urinary volume [22,23,24,25,26]. This osmotic diuresis increases electrolyte-free water clearance (CH_2_O), a key determinant of serum sodium concentration in SIADH.

Unlike loop diuretics, which disrupt the medullary concentration gradient, or vasopressin receptor antagonists, which directly inhibit vasopressin signaling, SGLT2 inhibitors act upstream without interfering with the counter-current mechanism or vasopressin receptors. This distinction is important in SIADH, where the primary defect is impaired excretion of electrolyte-free water due to persistent vasopressin activity [22,23,24,25,26].

The osmotic diuresis increases distal tubular flow and reduces the time available for vasopressin-mediated water reabsorption in the collecting duct. Functionally, this resembles an increase in effective free water clearance, a parameter central to SIADH pathophysiology. Through this mechanism, SGLT2 inhibitors reduce total body water in a gradual and physiologically targeted manner [22,23,24,25,26].

## 6. Clinical Evidence

Emerging clinical and mechanistic evidence has provided important insights into the potential role of sodium–glucose cotransporter-2 (SGLT2) inhibitors in the management of SIADH-associated hyponatraemia. Although the overall number of studies remains limited, several key randomised controlled trials, crossover studies, and mechanistic investigations have demonstrated consistent physiological effects supporting their therapeutic potential [27,28,29].

One of the most important clinical trials evaluating this approach was conducted by Refardt et al. in 2020 [28], who performed a randomised, double-blind, placebo-controlled study investigating the effects of empagliflozin in hospitalised patients with SIADH-associated hyponatraemia. This study included patients with confirmed SIADH and serum sodium concentrations below 130 mmol/L. Participants were randomised to receive empagliflozin 25 mg daily in addition to standard fluid restriction or placebo with fluid restriction. The results demonstrated that empagliflozin significantly increased serum sodium concentrations compared with placebo over a four-day treatment period. The improvement in sodium levels was accompanied by increased urinary volume and enhanced electrolyte-free water clearance, supporting the proposed osmotic diuretic mechanism. Importantly, the rate of sodium correction was gradual, and no cases of overly rapid correction or osmotic demyelination syndrome were observed. This study provided the first randomised clinical evidence supporting the efficacy and safety of SGLT2 inhibition in SIADH [27,28,29].

Further supporting evidence was provided by an earlier mechanistic study by Refardt et al. in 2017 [27], which examined the effects of empagliflozin in healthy volunteers with experimentally induced SIADH using desmopressin administration and controlled fluid intake. This study demonstrated that empagliflozin significantly increased urinary volume and promoted osmotic diuresis, even in the presence of persistent vasopressin activity. The increase in urinary output was accompanied by increased glucose excretion and reduced urine osmolality, confirming that SGLT2 inhibition can enhance renal water excretion independently of vasopressin receptor blockade. These findings provided important physiological proof-of-concept supporting the mechanistic rationale for SGLT2 inhibitor use in SIADH [27,28,29].

More recently, Refardt et al. conducted a randomised, double-blind, placebo-controlled crossover trial published in 2023, evaluating the effects of empagliflozin in outpatients with chronic SIADH. This study was particularly important as it examined longer-term outpatient management, which represents a major clinical challenge in SIADH. Patients received empagliflozin and placebo sequentially, allowing each participant to serve as their own control. The results demonstrated a statistically significant increase in serum sodium concentration during empagliflozin treatment compared with placebo. In addition, urinary volume increased and measures of free water clearance improved. Notably, empagliflozin was well-tolerated, with no significant increase in adverse events. This study provided important evidence supporting the potential role of SGLT2 inhibitors in the chronic outpatient management of SIADH [27,28,29].

In addition to these clinical trials, mechanistic studies have further clarified the physiological effects of SGLT2 inhibition relevant to SIADH. By promoting glucosuria, SGLT2 inhibitors create an osmotic gradient within the tubular lumen that limits passive water reabsorption in downstream nephron segments. This mechanism is particularly relevant in SIADH, where persistent vasopressin activity would otherwise promote excessive water reabsorption. The resulting increase in electrolyte-free water clearance leads to gradual reduction in total body water and improvement in serum sodium concentration [27,28,29].

Importantly, the magnitude of sodium correction observed in clinical trials has generally been modest, typically in the range of 3–5 mmol/L. However, even modest improvements in serum sodium can have significant clinical benefits, particularly in patients with chronic hyponatraemia, where gradual correction is desirable to minimise the risk of osmotic demyelination syndrome. The gradual and predictable nature of sodium correction observed with SGLT2 inhibitors may represent an important safety advantage compared with vasopressin receptor antagonists, which can produce rapid and sometimes excessive increases in serum sodium [27,28,29].

Despite these promising findings, several limitations must be considered. Most clinical studies conducted to date have involved relatively small sample sizes, limiting statistical power and generalisability. In addition, treatment durations have been relatively short, and long-term safety and efficacy data remain limited. Many studies have also excluded patients with severe hyponatraemia or significant comorbidities, and therefore the effectiveness of SGLT2 inhibitors in these higher-risk populations remains uncertain [27,28,29].

Importantly, no studies to date have evaluated hard clinical endpoints such as falls, fracture risk, cognitive outcomes, hospitalisation rates, or mortality. Available trials primarily assess short-term biochemical correction of serum sodium. Therefore, current evidence should be regarded as proof-of-concept rather than definitive evidence of long-term clinical benefit.

Another important consideration is that many patients in clinical trials received concurrent fluid restriction, which may have contributed to the observed improvements in serum sodium. The independent effect of SGLT2 inhibitors without fluid restriction requires further investigation. Furthermore, while empagliflozin has been the most extensively studied agent in this context, limited data are available for other SGLT2 inhibitors such as dapagliflozin, although their shared mechanism of action suggests similar physiological effects [27,28,29]. At present, direct clinical data evaluating dapagliflozin specifically in SIADH remain limited. Evidence is largely extrapolated from its established renal physiological effects and broader cardiovascular and renal outcome trials. Dedicated studies evaluating dapagliflozin in SIADH are required before conclusions regarding comparative efficacy can be drawn.

Overall, the available clinical evidence supports the concept that SGLT2 inhibitors can increase serum sodium concentration and enhance free water clearance in patients with SIADH. Their favourable tolerability profile, gradual correction of hyponatraemia, and mechanistically targeted action make them a promising therapeutic option. However, larger randomised controlled trials with longer follow-up are required to confirm these findings, establish optimal treatment protocols, and define their role within clinical practice [27,28,29].

## 7. Comparison with Existing Therapies

The management of SIADH-associated hyponatraemia remains challenging due to limitations associated with existing treatment options. SGLT2 inhibitors represent a mechanistically distinct approach, and their potential role is best understood in comparison with established therapies, including fluid restriction, vasopressin receptor antagonists, demeclocycline, loop diuretics, and urea [30,31,32,33,34,35,36,37,38,39,40].

Fluid restriction remains the recommended first-line therapy in most clinical guidelines. Its effectiveness is based on reducing free water intake below the kidney’s impaired excretory capacity. However, fluid restriction is frequently poorly tolerated, particularly in patients with chronic SIADH, due to persistent thirst driven by underlying pathophysiological mechanisms. Furthermore, its effectiveness is often limited in patients with high urine osmolality, where even strict fluid restriction may fail to produce adequate sodium correction. In contrast, SGLT2 inhibitors increase renal free water excretion independent of fluid intake, potentially offering a more effective and sustainable approach [30,31,32,33,34,35,36,37,38,39,40].

Vasopressin receptor antagonists, such as tolvaptan, directly target the underlying pathophysiology of SIADH by blocking V2 receptors in the collecting duct, thereby reducing aquaporin-2 channel insertion and promoting free water excretion. These agents are highly effective and can produce rapid increases in serum sodium concentration. However, their use is limited by several important factors, including high cost, the need for close monitoring, and safety concerns. Rapid overcorrection of serum sodium can occur, increasing the risk of osmotic demyelination syndrome. In addition, hepatotoxicity has been reported with prolonged use, necessitating liver function monitoring. In comparison, SGLT2 inhibitors produce more gradual sodium correction through osmotic diuresis, which may reduce the risk of overly rapid correction and associated complications [30,31,32,33,34,35,36,37,38,39,40].

Demeclocycline has historically been used in refractory SIADH due to its ability to induce nephrogenic diabetes insipidus by impairing collecting duct responsiveness to vasopressin. However, its use is limited by delayed onset of action, unpredictable response, and risk of nephrotoxicity, particularly in elderly patients or those with pre-existing renal impairment. In contrast, SGLT2 inhibitors have a more predictable mechanism of action and are associated with favourable renal outcomes in other clinical settings, including chronic kidney disease and heart failure [30,31,32,33,34,35,36,37,38,39,40].

Loop diuretics, often used in combination with oral sodium supplementation, act by inhibiting sodium reabsorption in the thick ascending limb of the loop of Henle, thereby disrupting the medullary concentration gradient and reducing water reabsorption. However, this approach may lead to excessive sodium loss and requires careful electrolyte monitoring. The evidence supporting this strategy is also limited. SGLT2 inhibitors differ fundamentally in their mechanism, acting in the proximal tubule to promote osmotic diuresis without directly disrupting the counter-current multiplication system [30,31,32,33,34,35,36,37,38,39,40].

Urea represents another therapeutic option that increases osmotic excretion of water by increasing renal solute load. While effective, its use is limited by poor palatability and patient acceptability, particularly in long-term treatment. SGLT2 inhibitors may offer similar osmotic effects while being more acceptable to patients, given their oral tablet formulation and established use in other conditions [30,31,32,33,34,35,36,37,38,39,40].

Taken together, SGLT2 inhibitors offer several potential advantages over existing therapies, including gradual and controlled correction of serum sodium, favourable tolerability, and mechanistic alignment with renal physiology. Rather than replacing existing treatments entirely, they may serve as a complementary or alternative option in selected patients, particularly in those who are unable to tolerate or respond to conventional therapies [30,31,32,33,34,35,36,37,38,39,40].

## 8. Safety and Adverse Effects of SGLT2 Inhibitors in SIADH

Although SGLT2 inhibitors are generally well-tolerated, their safety profile must be carefully considered when evaluating their potential role in the management of SIADH. These agents have been extensively studied in patients with diabetes, heart failure, and chronic kidney disease, providing valuable insights into their safety [41,42,43,44,45,46,47].

One of the most important potential adverse effects is volume depletion. By promoting osmotic diuresis, SGLT2 inhibitors increase urinary fluid loss, which may lead to hypotension, dizziness, and dehydration, particularly in elderly patients or those with reduced oral intake. Careful patient selection and monitoring are therefore essential when initiating therapy in individuals with SIADH, who may already have altered fluid balance [41,42,43,44,45,46,47].

Another important consideration is the risk of euglycaemic diabetic ketoacidosis, a rare but serious complication associated with SGLT2 inhibitors. Although this risk is primarily observed in patients with diabetes, caution is warranted when using these agents in non-diabetic individuals, particularly in situations of prolonged fasting, acute illness, or reduced carbohydrate intake [41,42,43,44,45,46,47].

SGLT2 inhibitors are also associated with an increased risk of genital infections, including fungal infections, due to glucosuria. These infections are generally mild and manageable but should be discussed with patients prior to initiation of therapy [41,42,43,44,45,46,47].

Importantly, clinical trials evaluating empagliflozin in SIADH have demonstrated favourable safety profiles, with no significant increase in serious adverse events compared with placebo. The gradual increase in serum sodium observed with SGLT2 inhibitors may represent an important safety advantage, reducing the risk of overly rapid correction and osmotic demyelination syndrome [41,42,43,44,45,46,47].

Overall, while SGLT2 inhibitors appear to be well-tolerated, careful patient selection, monitoring of fluid status, and awareness of potential adverse effects are essential to ensure safe use in SIADH [41,42,43,44,45,46,47].

## 9. Clinical Positioning and Treatment Algorithm

SGLT2 inhibitors may be particularly valuable in patients with chronic SIADH who fail fluid restriction, are intolerant of vaptans, or are at risk of nephrotoxicity from demeclocycline. They may be especially suited to outpatient management where gradual and sustained correction of serum sodium is desirable. SGLT2 inhibitors may also be particularly useful in patients requiring long-term outpatient therapy where fluid restriction is ineffective or poorly tolerated. Patients with preserved renal function, stable haemodynamics and absence of significant hypovolaemia represent ideal candidates. Caution is warranted in individuals at risk of volume depletion or ketoacidosis. Importantly, the gradual nature of sodium correction observed in trials suggests a lower risk of osmotic demyelination compared with more aggressive therapies [36,37,38,39,40].

It is important to note that current international guidelines for the management of SIADH do not include SGLT2 inhibitors as a recommended therapy. Their use in this context remains off-label and investigational.

## 10. Future Directions

Despite promising early evidence, the role of SGLT2 inhibitors in the management of SIADH remains incompletely defined, and several important questions require further investigation [48,49,50].

First, larger randomised controlled trials are needed to confirm the efficacy and safety of SGLT2 inhibitors in diverse patient populations, including those with more severe hyponatraemia and significant comorbidities. Most existing studies have involved relatively small sample sizes and short follow-up durations, limiting the ability to assess long-term outcomes [48,49,50].

Second, optimal treatment protocols remain unclear. Future studies should investigate appropriate dosing strategies, duration of therapy, and whether SGLT2 inhibitors should be used as monotherapy or in combination with other treatments such as fluid restriction or sodium supplementation [48,49,50].

Third, further research is needed to identify patient populations most likely to benefit from SGLT2 inhibitor therapy. This may include patients with chronic SIADH, those who are intolerant of existing therapies, or individuals at high risk of complications related to hyponatraemia [48,49,50].

In addition, comparative studies directly evaluating SGLT2 inhibitors against established therapies such as tolvaptan would provide valuable information regarding their relative efficacy, safety, and cost-effectiveness [48,49,50].

Mechanistic studies may also help clarify the precise physiological effects of SGLT2 inhibition in SIADH and improve understanding of their impact on renal water handling [48,49,50].

Finally, real-world observational studies will be important to assess long-term safety and effectiveness in routine clinical practice [48,49,50].

Taken together, these future investigations will help define the optimal role of SGLT2 inhibitors within treatment algorithms for SIADH and determine whether they may become part of standard clinical management [48,49,50].

## 11. Conclusions

SIADH remains a common and clinically significant cause of hyponatraemia, associated with substantial morbidity and healthcare burden. Despite advances in understanding its pathophysiology, management remains challenging due to limitations associated with existing therapies, including poor tolerability, safety concerns, and variable efficacy [1,2,3,4,5,6,27,28,29].

SGLT2 inhibitors represent a novel and physiologically rational therapeutic approach based on their ability to promote glucosuria-induced osmotic diuresis and enhance electrolyte-free water clearance. Emerging clinical evidence, including randomised controlled trials and mechanistic studies, has demonstrated that these agents can produce modest but clinically meaningful improvements in serum sodium concentrations in patients with SIADH [1,2,3,4,5,6,27,28,29].

Importantly, their mechanism of action differs fundamentally from traditional therapies, offering gradual correction of hyponatraemia without directly interfering with vasopressin signalling or disrupting renal concentrating mechanisms. Their favourable safety profile, oral administration, and established use in other clinical settings further support their potential role [1,2,3,4,5,6,27,28,29].

While current evidence is promising, larger and longer-term clinical trials are required to confirm their efficacy, define optimal treatment protocols, and determine their precise position within treatment algorithms [1,2,3,4,5,6,27,28,29].

**Figure 1:**
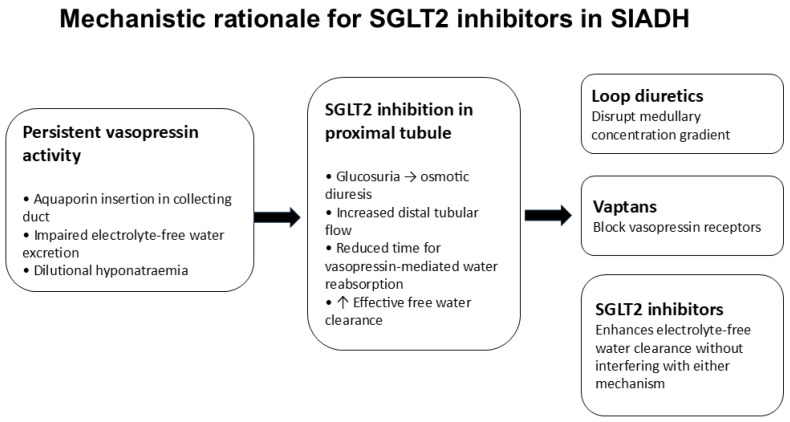
Mechanistic rationale for the use of SGLT2 inhibitors in SIADH. SGLT2 inhibition promotes glucosuria-driven osmotic diuresis, increases distal tubular flow and enhances effective free water clearance, offering a physiologically distinct approach compared with loop diuretics and vasopressin receptor antagonists [12,13,14,15,16,22,23,24,25,26,27,28,29].

In conclusion, SGLT2 inhibitors represent a mechanistically rational and promising therapeutic approach in SIADH-associated hyponatraemia. However, current evidence remains limited to small, short-term studies focused primarily on biochemical endpoints. Their use should therefore be considered investigational pending confirmation from larger, long-term outcome-driven trials and before integration into formal clinical guideline recommendations (Figure 1 and Table 1).

## Figures and Tables

**Table 1:** Summary of clinical studies evaluating SGLT2 inhibitors in SIADH.

Study	Population	Design	Intervention	Outcome	Key Result
Refardt 2020 [28]	Inpatient SIADH	RCT	Empagliflozin	Serum Na change	Significant rise via free water clearance
Refardt 2017 [27]	Induced SIAD model	Mechanistic study	Empagliflozin	Urinary volume	Increased osmotic diuresis
Refardt 2023 [29]	Chronic SIADH outpatients	Crossover RCT	Empagliflozin	Na and urine output	Sustained improvement in sodium and fluid balance

## Data Availability

No new data were created or analysed in this study. Data sharing is not applicable to this article.
